# Serosurveillance and Postvaccination Seromonitoring of Peste Des Petits Ruminants in Small Ruminants in Central Gondar Zone, Northwest Ethiopia

**DOI:** 10.1155/tbed/3319431

**Published:** 2026-09-28

**Authors:** Firdyawukal Abuhay Tafere, Mastewal Birhan Atanaw, Enyiew Alemnew Alamerew, Melkie Dagnaw Fenta, Genetu Kassahun Berie, Kefale Ambachew Shiferaw, Bemrew Admassu Mengistu, Anmaw Shite Abat

**Affiliations:** ^1^ Department of Veterinary Pathobiology, College of Veterinary Medicine and Animal Science, University of Gondar, Gondar, Ethiopia, uog.edu.et; ^2^ Amhara Agricultural Research Institute, Debre Birhan Agricultural Research Centre, Debre Birhan, Ethiopia, arari.gov.et; ^3^ Department of Clinical Veterinary Medicine, College of Veterinary Medicine and Animal Science, University of Gondar, Gondar, Ethiopia, uog.edu.et; ^4^ Institute of Biotechnology, Department of Medical Biotechnology, University of Gondar, Gondar, Ethiopia, uog.edu.et; ^5^ Department of Biomedical Sciences, College of Veterinary Medicine and Animal Science, University of Gondar, Gondar, Ethiopia, uog.edu.et

**Keywords:** antibody immunity, eradication, risk-based vaccination strategy, seroprevalence

## Abstract

Peste des petits ruminants (PPR) is a highly contagious transboundary viral disease of small ruminants caused by the PPR virus (PPRV). Despite Ethiopia’s goal of eradicating PPR by 2027 through a risk‐based vaccination strategy, the disease remains endemic, with 554 outbreaks reported nationwide. This cross‐sectional study was conducted in the North Gondar Zone, Ethiopia, between June 2024 and February 2025 to evaluate antibody responses in vaccinated small ruminants and estimate PPRV seroprevalence in unvaccinated flocks. A multistage cluster sampling design was used to select the study sites, while individual animals were selected using a haphazard sampling approach to approximate random sampling. A total of 600 serum samples were collected, including 300 from vaccinated and 300 from unvaccinated small ruminants; samples from vaccinated animals were obtained ~28 days postvaccination. All samples were tested for anti‐PPRV antibodies using competitive enzyme‐linked immunosorbent assay (c‐ELISA). Overall individual‐level seropositivity in the vaccinated population was 75%, comprising 75.73% in sheep and 72.1% in goats, serving as a proxy measure of flock immunity. In unvaccinated animals, PPRV seroprevalence was 14.33% (95% confidence interval [CI]: 10.6%–18.82%). Multivariable logistic regression identified age and flock size as the main risk factors influencing seropositivity. Adult animals were 2.89 times as likely to be seropositive as young animals (95% CI: 1.17–7.15; *p* = 0.022), and animals in medium‐sized flocks were more likely to test positive than those in small flocks (AOR = 2.26; 95% CI: 1.11–4.63; *p* = 0.026). No significant differences were observed across districts, species, sex, production systems, newly introduced animals, or animal movement (*p*  > 0.05). The observed seropositivity was slightly below the commonly used 80% population immunity target associated with reducing transmission under the PPR eradication framework. The detection of PPRV antibodies in unvaccinated animals indicates previous exposure to PPRV and highlights the need for strengthened surveillance to assess whether active virus circulation persists in the area. To achieve effective control and eventual eradication of PPR, intensified vaccination programs combined with active surveillance are needed. Furthermore, refining vaccination strategies in alignment with global and national eradication plans is essential to establish PPR‐free zones in these districts.

## 1. Introduction

Peste des petits ruminants (PPR) is a highly contagious viral disease affecting sheep and goats, caused by PPR virus (PPRV), a member of the genus *Morbillivirus* within the family Paramyxoviridae [1]. It affects small ruminant populations across Africa, the Middle East, and Asia, resulting in high morbidity, mortality, and reduced productivity. These impacts threaten livelihoods, food security, and income, particularly for subsistence farmers who rely on sheep and goats for meat, milk, and trade [2–4].

To mitigate its impact, the Food and Agriculture Organization (FAO) and the World Organization for Animal Health (WOAH) launched a global PPR control initiative in 2015, targeting eradication by 2030 [5]. In alignment with this initiative, the Ethiopian Ministry of Agriculture, in collaboration with FAO, aims to eradicate PPR nationally by 2027 through risk‐based vaccination campaigns, strengthened surveillance, and postvaccination monitoring [6]. Despite these efforts, PPR remains a major challenge, with over 70 countries still reporting outbreaks [7]. In Ethiopia, outbreaks continue even with the implementation of risk‐based vaccination strategies, with 554 PPR outbreaks recorded nationwide between 2018 and 2022 [6, 7].

Postvaccination seromonitoring and epidemiological studies are essential for evaluating antibody responses, identifying gaps in vaccination coverage, and understanding disease prevalence and transmission dynamics [7, 8]. In Central Gondar, limited evidence on the effectiveness of the risk‐based vaccination program constrains proper assessment and timely outbreak response. This information gap hinders the evaluation of postvaccination immunity, detection of ongoing virus circulation, and identification of factors associated with PPRV infection, all of which are necessary for evidence‐based decision‐making and improved vaccination strategies. Therefore, this study aimed to assess antibody responses in vaccinated small ruminants, estimate PPRV seroprevalence in unvaccinated flocks, and identify animal‐level risk factors associated with seropositivity. The findings provide evidence to evaluate the current vaccination program and support strengthened PPR control and eradication efforts in the region.

## 2. Materials and Methods

### 2.1. Study Area

The study was conducted in the East Dembia and Gondar Zuria districts of the North Gondar Zone, Amhara Regional State, Northwest Ethiopia. Geographically, the area lies between 37°10^′^E–37°40^′^E longitude and 12°10^′^N–12°40^′^N latitude (Figure 1). The districts are characterized by mixed crop–livestock farming systems, with small ruminant production playing an important economic role. The Amhara Region hosts large populations of small ruminants, estimated at 10,267,358 sheep and 6,808,301 goats [9]. Within the North Gondar Zone, ~507,316 sheep and 557,070 goats are raised. The East Dembia district has about 51,151 sheep and 14,195 goats, while Gondar Zuria contains an estimated 86,356 sheep and 67,696 goats. PPR outbreaks occur frequently in these districts, prompting annual vaccination campaigns conducted from September to February. Increased animal movement and communal grazing after the harvest season, particularly from December onward, are associated with a higher risk of disease transmission.

**Figure 1:**
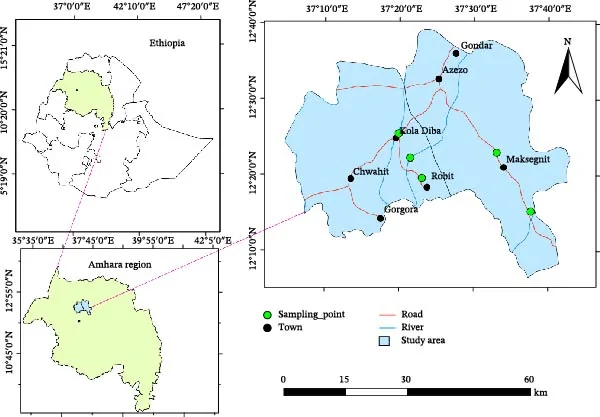
Map of the study area.

### 2.2. Study Population

The study population comprised small ruminants, specifically sheep and goats, maintained under field conditions within a mixed crop–livestock farming system. Only clinically healthy animals were included. The animals were managed under extensive production systems, where individual household flocks commonly intermingle at shared grazing and watering points.

### 2.3. Study Design

A cross‐sectional serosurveillance study combined with a postvaccination seromonitoring component was conducted from June 2024 to February 2025.

### 2.4. Sample Size Determination

The sample size was calculated using a standard formula described in a previous study [10]. In the absence of comparable data for the study area, an expected PPRV seroprevalence of 50% and a 50% postvaccination seromonitoring response were assumed to obtain the maximum required sample size. A standard error of 0.05 was used to estimate the number of clusters required for the study. Based on these assumptions, 27 clusters were determined for both seromonitoring and serosurveillance components. Each objective required ~300 samples, resulting in a total sample size of 600 animals. The formula used for the sample size calculation is presented as follows:

n=gc=P1−PDSE2.

In this formula, ’*n*’ represents the sample size, **’p’** is the expected prevalence, ’*D*’ is the design effect, ’SE**’** stands for standard error, **’g’** is the average number of animals sampled per cluster, and **’c’** is the number of clusters (in this case, villages). The design effect (**D**) was calculated using the following formula:

D=1+g --1 ICC.

The intracluster correlation coefficient (ICC), which reflects the similarity of individuals within a cluster, is commonly reported to range between 0.05 and 0.2 in livestock infectious disease studies, including small‐ruminant epidemiological investigations [11]. In the absence of location‐specific estimates, a conservative ICC value of 0.2 was assumed for each village to avoid underestimating the required sample size. With an average of 11 serum samples (*g*) collected per village, the design effect was calculated to be *D* = 3. A total of 600 serum samples were collected, comprising 300 samples for serosurveillance and 300 for postvaccination seromonitoring. Sample collection was conducted with the consent of animal owners and based on herd accessibility.

### 2.5. Sampling Strategy

A multistage cluster sampling design with a nonprobability component was employed in the East Dembia and Gondar Zuria districts of the Amhara Region, Ethiopia. These districts were purposively selected due to their high small‐ruminant populations, making them suitable for evaluating vaccination outcomes under routine field conditions. Peasant associations (PAs, locally known as kebeles) were first selected, followed by selection of villages and animals within each PA. For the postvaccination seromonitoring component, PAs where vaccination had already been conducted, Sufankara and Guranba in East Dembia and Maksegnit and Enferaze in Gondar Zuria, were included. For the serosurveillance component, neighboring unvaccinated PAs, Robit and Kola Diba in East Dembia and Adeno‐Garno and Layeye in Gondar Zuria were selected. In total, eight PAs comprising 27 villages were conveniently chosen based on vaccination timing, accessibility, and logistical feasibility. Within villages, animals were selected using a haphazard approach to approximate random sampling. This nonprobability selection was necessary due to the absence of formal sampling frames, dispersed households, communal grazing practices, and other practical constraints of extensive production systems. Only clinically healthy sheep and goats older than 6 months were included as younger animals may carry maternal antibodies that could interfere with serological results.

### 2.6. Sample Collection

Approximately 5 mL of blood was collected from selected animals for postvaccination seromonitoring (28 days after vaccination to assess antibody responses) and serosurveillance from unvaccinated animals using direct jugular venipuncture with sterile Vacutainer systems. The animals were manually restrained during sampling. Blood samples were allowed to clot at room temperature for 30–60 min before transport to the laboratory as sampling sites were distant and lacked immediate access to electricity. Samples were then centrifuged at 3000 rpm at the University of Gondar Veterinary Microbiology Laboratory to separate the serum, which was stored at –20°C until transport to the National Veterinary Institute (NVI) in Debre Zeit, Ethiopia, for laboratory testing (Annex II).

During sample collection, flock sizes were categorized as small (<10 animals), medium (10–20 animals), or large (>20 animals) following previous classifications [12]. The age of each animal was estimated through consultation with its owner (Annex III) and grouped into two categories: young (6–18 months) and adult (>18 months) [13]. Epidemiological information on potential PPR risk factors, including location, vaccination history, species, sex, age, flock size, and animal movements, was collected using a standardized sample collection form (Annex I).

### 2.7. Serological Examination

Serological analysis for PPRV antibodies was conducted at the NVI in Debre Zeit, Ethiopia. The assay employed was the ID Screen PPR Competition enzyme‐linked immunosorbent assay (ELISA) kit (IDvet Screen PPR Competition Kit, 310 Rue Louis Pasteur, 34790 Grabels, France), a validated tool known for its high sensitivity of 94.5% and specificity of 99.4% [14]. This competitive ELISA (c‐ELISA) method specifically identifies antibodies against the PPRV nucleoprotein. We determined sample positivity or negativity by measuring the optical density (OD) at 450 nm with an ELISA plate reader and then computing the sample‐to‐negative (S/N) ratio as per the manufacturer’s instructions, as shown in (Annex IV and Annex V).

SN=ODSODNC×100,

where ODS = OD of the sample, and ODNC = OD of the negative control. Test samples that showed S/N values of less than or equal to 50% were considered positive; those with S/N values of greater than 50% but less than or equal to 60% were considered doubtful, while test samples that showed S/N values above 60% were considered negative. Samples with doubtful results were retested, and only clearly positive or negative samples were included in the statistical analyses.

### 2.8. Data Management and Statistical Analysis

Data from the study were compiled and organized in an MS Excel spreadsheet and analyzed using STATA Version 17. Descriptive statistics were used to calculate the seroprevalence of PPRV antibodies in the study areas. A null mixed‐effects logistic regression model with PA as a random intercept was initially fitted to assess the clustering of PPRV seropositivity. The ICC was estimated using the latent‐variable approach for logistic mixed models, and a likelihood ratio test was performed to compare the mixed‐effects and ordinary logistic regression models. As the between‐PA variance and ICC were negligible, and the likelihood ratio test indicated no evidence of significant clustering, subsequent risk factor analyses were performed using ordinary logistic regression. To identify risk factors associated with the presence of PPRV antibodies, univariable logistic regression was performed for variables including age, breed, species, and sex. Variables with a *p*‐value < 0.25 in the univariable analysis were included in the multivariable logistic regression model. A forward stepwise selection approach was used, adding variables one at a time, starting with the most significant, and a backward stepwise procedure was also employed to refine variable selection. Spearman rank correlations were used to assess the relationships between independent variables. If the correlation coefficient between the two variables was ≥0.7, only one variable was retained in the multivariable model. All possible two‐way interactions between variables in the final model were evaluated. Potential confounding was assessed during model building by examining changes in beta estimates when variables were added. Variables causing >30% change in beta estimates were considered confounders [15]. Statistical significance was determined at a *p*‐value < 0.05, and 95% confidence intervals (CI) were reported for all estimates.

### 2.9. Ethical Consideration

This study was conducted after being evaluated and approved by the Institutional Review Board of the University of Gondar. This study followed the protocols of the National Research Ethics Review Guideline, formulated by the Ministry of Science and Technology of the Federal Democratic Republic of Ethiopia, in 2014. Sheep and goat populations were treated with best practices of veterinary care (Ref: CVMASc /UOG/RERC/22/10/2024), as indicated (Annex VI). Before any animals were included in the study, informed verbal consent was obtained from the sheep’s owners. They were fully informed about the research’s nature, the procedures involved, and the potential impacts on their animals. The owners were also made aware of their right to withdraw their animals from the study at any point. The study prioritized animal welfare, and every effort was made to minimize potential discomfort or harm to the animals throughout the study.

## 3. Results

### 3.1. Antibody Immunity in Vaccinated Small Ruminants

A total of 300 serum samples were collected from two districts, of which 225 tested positive for PPRV antibodies, corresponding to an overall individual‐level seropositivity rate of 75%. At the district level, East Dembia exhibited a slightly higher seropositivity rate (77.03%), whereas Gondar Zuria showed a marginally lower seropositivity rate (73.03%). Among the (PAs, Maksegnit recorded the highest seropositivity rate (81.82%), while Sufankara had the lowest flock‐level seropositivity rate against PPRV (71.7%) (Table 1). Of the seropositive animals, 181 (75.73%) were sheep and 44 (72.1%) were goats (Table 2). However, these seropositivity rates remain below the commonly used 80% population immunity target adopted within the PPR eradication framework.

**Table 1:** Antibody immunity against PPRV in vaccinated small ruminants across study areas.

Districts	Peasant associations	Number of sampled	Number of positives	Prevalence (%)	CI (95%)	
					Lower	Upper
East Dembia	Guramba	95	76	80	70.54	87.51
	Sufankara	53	38	71.7	57.65	83.21
	Subtotal	148	114	77.03	69.41	83.54
Gondar Zuria	Enferanze	97	71	73.2	63.24	81.69
	Maksegnit	55	45	81.8	69.1	90.92
	Subtotal	152	111	73.03	65.24	79.9

Overall		300	225	75.0	69.7	79.8

**Table 2:** Univariable logistic regression model for potential factors influencing the immune response to PPRV vaccination in vaccinated small ruminants.

Risk factors	Category	Number of tested	Number of positive	Prevalence (%)	COR	95% CI		*p*‐Value
						Lower	Upper	
District	East Dembia	148	114	77.03	1.24	0.73	2.09	0.42
	Gondar Zuria^a^	152	111	73.03	1	—	—	—
Species	Sheep	239	181	75.73	1.21	0.64	2.27	0.56
	Goat^a^	61	44	72.13	1	—	—	—
Sex	Female	104	73	70.2	1	—	—	—
	Male	196	152	77.6	1.47	0.86	2.51	0.16
Age	Young^a^	124	85	68.6	1	—	—	—
	Adult	176	140	79.6	1.78	1.05	3.02	0.03
Flock size	Small	154	116	75.32	1.09	0.64	1.86	0.32
	Medium^a^	133	98	73.7	1	—	—	—
	Large	13	11	84.62	1.96	0.42	9.30	0.4

*Note:* %, percentage.

Abbreviations: CI, confidence interval; COR, crud odds ratio.

^a^Reference.

The null mixed‐effects logistic regression model showed negligible between‐PA variation in postvaccination immune status (*σ* ^2^ = 2.52 × 10^−32^; ICC ≈ 0.00), and the likelihood ratio test indicated no evidence of significant clustering. Univariable logistic regression analysis revealed significant differences (*p* < 0.05) in immune status among the age groups, with adults (79.6%, CI = 1.05–3.02) being nearly twice as likely to be seropositive as young animals (68.6%). In contrast, no significant differences in seropositivity rates were observed across species, sexes, flock sizes, or among the study districts (Table 2).

In the multivariable model (*p* < 0.25), age and sex were selected as potential factors associated with PPRV seropositivity (Table 3). Spearman rank correlation analysis showed no significant correlation between the independent variables. Confounding was assessed during model development, but no confounders were identified. All two‐way interactions were tested and found to be nonsignificant. Following variable selection, the final logistic regression model retained age as the only independent predictor associated with PPRV seropositivity (OR 1.78, 95% CI: 1.05–1.08, *p* = 0.03) (Table 3). Because no other variables remained in the final model, the estimated effect size for age was identical to that obtained from the univariable analysis.

**Table 3:** Final logistic regression model of factors associated with PPRV seropositivity after variable selection.

Risk factors	Category	Adjusted odds ratio	*p*‐Value	CI (95%)	
				Lower	Upper
Age	Young ^∗^	1	—	—	—
	Adult	1.78	0.03	1.05	3.02

*Note:*  ^∗^ indicates the reference for comparison with their scientific context.

### 3.2. Seroprevalence and Associated Risk Factors of PPR in Unvaccinated Flocks

A total of 300 samples were collected from two districts. Of these, 43 tested positive for PPRV antibodies, resulting in an overall seroprevalence of 14.33%. The highest district‐level seroprevalence was observed in East Dembia (15.8%), whereas Gondar Zuria recorded the lowest (12.8%). At the PA level, Robit had the highest seroprevalence (17.7%), while Layeye had the lowest (12.2%) (Table 4).

**Table 4:** Seroprevalence of PPR in unvaccinated flocks across the study district and PAs.

Districts	Peasant associations	Number of tested	Number of positive	Prevalence (%)	CI (95%)	
					Lower	Upper
East Dembia	Kola Diba	73	10	13.7	6.8	18.82
	Robit	79	14	17.7	10.04	27.9
	Subtotal	152	24	15.8	10.4	22.6
Gondar Zuria	Layeye	74	9	12.16	5.71	21.84
	Adeno‐Garno	74	10	13.51	6.68	23.45
	Subtotal	148	19	12.84	7.91	19.32

Overall		300	43	14.33	10.6	18.82

The null mixed‐effects logistic regression model indicated negligible between‐PA variation in PPRV seropositivity (*σ* ^2^ = 2.52 × 10^−32^; ICC ≈ 0.00), and the likelihood ratio test showed that the mixed‐effects model did not provide a better fit than the ordinary logistic regression model. Among unvaccinated small ruminants, seroprevalence ranged from 6.9% to 17.4% across age groups and from 9.9% to 19.7% across flock sizes. Univariable logistic regression showed that both age and flock size were significantly associated with PPRV seropositivity (*p*  < 0.05), with adults and animals from medium‐sized flocks having higher odds of seropositivity than their respective reference groups (95% CI: 1.15–6.99 and 1.09–4.52, respectively). No significant associations were found for district, species, sex, production system, newly introduced animals, or animal movement (*p* > 0.05) (Table 5).

**Table 5:** Univariable logistic regression of PPR seropositivity in unvaccinated sheep and goats in the study area.

Risk factors	Category	Number of tested	Number of positive	Prevalence (%)	Adjusted odds ratio	*p*‐Value	Confidence interval (95%)	
							Lower	Upper
District	East Dembia	152	24	15.8	1.27	0.47	0.67	2.44
	Gondar Zuria^a^	148	19	12.84	1	—	—	—
Species	Sheep	182	28	15.4	1.25	0.52	0.64	2.45
	Goat^a^	118	15	12.7	1	—	—	—
Sex	Female	113	17	15.04	1.1	0.79	0.57	2.13
	Male^a^	187	26	13.9	1	—	—	—
Age	Young^a^	87	6	6.9	1	—	—	—
	Adult	213	37	17.4	2.84	0.02	1.15	6.99
Flock size	Small^a^	141	14	9.9	1	—	—	—
	Medium	122	24	19.7	2.22	0.03	1.09	4.52
	Large	37	5	13.5	1.42	0.53	0.48	4.23
Production system	Extensive^a^	219	28	12.8	1	—	—	—
	Semi‐intensive	81	15	18.5	1.55	0.21	0.78	3.08
New animal introduces	Yes	126	21	16.7	1.38	0.328	0.72	2.64
	No^a^	174	22	12.6	1	—	—	—
Animal movement	Low	140	22	15.7	1.23	0.52	0.65	2.36
	High^a^	160	21	13.1	1	—	—	—

^a^Reference.

In the multivariable model (using a *p*‐value threshold of <0.25 for inclusion), age, flock size, and production system were selected as potential factors associated with PPRV infection. Spearman rank correlation analysis revealed no significant correlation between the independent variables. Confounding was assessed during model development, but no confounders were identified. All two‐way interactions were tested and found to be nonsignificant. The final multivariable logistic regression model identified age and flock size as the most critical risk factors influencing differences in PPR seropositivity. Specifically, adult animals were 2.89 times more likely to be seropositive for PPRV infection compared to young animals (95% CI: 1.17–7.15; *p* = 0.022). Additionally, sheep and goats in medium‐sized flocks were more likely to test seropositive for PPR compared to those in small flocks (AOR = 2.26; 95% CI: 1.11–4.63; *p* = 0.026) (Table 6).

**Table 6:** Multivariable logistic regression for potential factors of PPR seropositivity in small ruminants.

Risk factors	Category	AOR	*p*‐Value	CI (95%)	
				Lower	Upper
Age	Young ^∗^	1	—	—	—
	Adult	2.89	0.022	1.17	7.15
Flock size	Small ^∗^	1	—	—	—
	Medium	2.26	0.026	1.11	4.63
	Large	1.48	0.488	0.49	4.45

*Note:*  ^∗^ indicates the reference for comparison with their scientific context.

## 4. Discussion

### 4.1. Flock Immunity

PPR, commonly referred to as goat plague, is a highly contagious viral disease that poses substantial socioeconomic threats worldwide [16]. Among available control measures, vaccination remains the most effective and practical strategy, particularly in resource‐limited countries such as Ethiopia [17]. Although movement control can contribute to disease reduction, its implementation is challenging under extensive livestock production systems [17, 18]. Consequently, coordinated regional and international initiatives aim to eradicate PPR by 2030 [19–23]. Placing vaccination and systematic postvaccination evaluation (PVE) at the center of global eradication efforts is important. Given its large small‐ruminant population and continued PPR risk, the Amhara National Regional State represents an important area for evaluating vaccination effectiveness and supporting evidence‐based eradication strategies.

Within the global eradication framework, seromonitoring is emphasized as a key component of PVE, enabling assessment of population‐level antibody immunity and vaccination performance under local epidemiological conditions [22]. Accordingly, the present study assessed the postvaccination immune status of sheep and goats in the East Dembia and Gondar Zuria districts of the North Gondar Zone, where such an evaluation had not previously been conducted. The findings provide important evidence regarding the effectiveness of vaccination campaigns and identify potential gaps requiring targeted interventions to improve PPR control.

The present study demonstrated an overall individual‐level postvaccination seropositivity rate of 75% (95% CI: 69.7%–79.8%), indicating a substantial vaccine‐induced antibody response among vaccinated small ruminants. Although seropositivity is commonly used as an indicator of population immunity, it should not be interpreted as equivalent to complete protective immunity at either the individual or the flock level. Therefore, these findings should be interpreted as evidence of antibody response following vaccination rather than absolute protection against infection. Nevertheless, the observed seropositivity level was close to the ≥80% population immunity target recommended by the PPR Global Control and Eradication Strategy (PPR‐GCES) as a benchmark associated with reducing virus transmission [22, 23]. This suggests progress toward eradication objectives while emphasizing the need for sustained vaccination, surveillance, and monitoring to address the remaining immunity gaps.

The observed postvaccination seropositivity level was comparable with reports from other field‐based studies, including 76.66% in Sekela District, Ethiopia [24] and ~73.4% in India [8]. However, it was higher than immunity levels reported in Metema (68%) [25], Awash Fentale District (61%) shortly after vaccination [26], and North Shewa, Ethiopia (65.35%) [27]. The previously reported district‐level PPR seroprevalence of 48.42% in small ruminants (44.7% in sheep and 51.21% in goats) in Odisha [25]. Similarly, mass vaccination campaigns in Somalia increased the seroprevalence from 62% to 72% [28]. A wide range of seropositivity levels (21%–74%) reported from different regions of India has been attributed to differences in vaccination frequency, coverage, and implementation strategies [29]. In contrast, higher seropositivity levels (up to 96%) reported under experimental conditions [30] should be interpreted cautiously as controlled studies minimize the logistical, management, and operational challenges commonly encountered under field conditions. Therefore, differences among studies may reflect variation in vaccination coverage, timing of postvaccination assessment, vaccine delivery practices, cold chain maintenance, and field‐level implementation capacity.

At the district level, East Dembia showed a slightly higher postvaccination seropositivity rate (77.03%) compared with Gondar Zuria (73.03%). Similarly, Maksegnit recorded the highest seropositivity among PAs (81.82%), whereas Sufankara had the lowest (71.7%). However, these differences were not statistically significant, and the null mixed‐effects logistic regression model indicated negligible between‐PA variation (ICC ≈ 0.00). Therefore, the overall seropositivity estimate of 75% provides the most appropriate summary indicator of antibody immunity under the study conditions. Although no significant spatial variation was detected, maintaining adequate population immunity remains challenging under field conditions. Factors such as inadequate vaccine handling, particularly cold chain interruptions in remote areas, may reduce vaccine effectiveness [27]. Moreover, rapid population turnover due to births, sales, and slaughter can decrease population immunity between vaccination campaigns [28]. These challenges highlight the importance of repeated vaccination campaigns, strengthened vaccine delivery systems, and routine postvaccination monitoring to sustain sufficient population immunity during the eradication process.

Age was identified as the only independent factor significantly associated with postvaccination seropositivity. Adult animals were ~1.78 times more likely to be seropositive than young animals (AOR = 1.78; 95% CI: 1.05–3.02). This association may be explained by the greater likelihood of cumulative exposure to vaccination and PPRV antigens over time, whereas younger animals may exhibit lower antibody responses due to interference from maternally derived antibodies. The influence of age on seropositivity highlights the importance of considering population age structure when evaluating vaccination outcomes and designing effective immunization strategies. Similar age‐related differences in PPRV antibody status have been reported previously [27, 30]. Where older animals generally showed higher serological responses, likely due to increased opportunities for vaccination and/or previous exposure to PPRV antigens. In contrast, sex, flock size, and district were not significantly associated with antibody immunity, suggesting that these factors had limited influence on the serological response under the conditions of this study.

### 4.2. Seroprevalence and Associated Risk Factors

An effective surveillance system capable of detecting PPRV circulation, particularly among unvaccinated populations, is a critical component of the PPR eradication program [23]. Such surveillance is essential for accurately interpreting PVE results and distinguishing vaccination‐associated immunity from naturally acquired exposure. In the present study, unvaccinated sheep and goats from East Dembia and Gondar Zuria districts showed an overall PPRV antibody seroprevalence of 14.33% (95% CI: 10.6–18.82), indicating previous exposure to PPRV among susceptible small ruminants in the study area. The detection of antibodies in unvaccinated animals suggests a natural exposure to PPRV, which may contribute to the maintenance of susceptible populations and potential virus circulation. However, seropositivity alone does not confirm active virus circulation; therefore, further surveillance incorporating virus detection methods is required to determine ongoing transmission. Following natural infection, small ruminants generally develop long‐lasting immunity, which may reduce the proportion of susceptible animals in endemic settings [31].

The seroprevalence observed in the present study (14.33%) was higher than that reported in reports from several Ethiopian regions. For example, lower seroprevalence levels were reported in Southwest Ethiopia (2.1%) [12], Sekela District of the West Gojjam Zone, Amhara Region (3.61%) [24], and Mecha District of the same region (3.70%) [32]. In contrast, higher seroprevalence levels have been reported in several Ethiopian settings, including North Shewa, Amhara Region (56.17% in small ruminants; [33], 49.3% in sheep [34]). Eastern Shewa and Arsi zones of Oromia Region (48.43%) [35], South Omo Zone of Southern Ethiopia (48.43%) [36], and selected districts of Afar Region (60.15%) [37]. Additionally, studies conducted under the pastoral and agropastoral production systems reported seroprevalence levels of 7.3% in sheep and 42% in goats [38]. The current finding was relatively comparable with the overall seroprevalence reported from the Amhara Region (18.3%) [39], suggesting a similar level of natural PPRV exposure in some production systems. The observed differences in seroprevalence among studies may be explained by variations in vaccination history, livestock production systems, management practices, sample size, geographical location, seasonal factors, host population density, animal movement patterns, disease control interventions, and ecological conditions. These factors can influence contact rates between susceptible animals and infected populations, thereby affecting the likelihood of PPRV exposure and the observed serological patterns across regions.

Age was identified as a significant risk factor for PPRV seropositivity in the present study. Adult small ruminants had a 2.89‐fold higher likelihood of being seropositive compared that of young animals. The higher seroprevalence observed among adults may be attributed to cumulative exposure to PPRV over their lifetime, increasing the probability of previous infection. Similar associations between age and PPRV seropositivity have also been reported in previous studies [40–43]. This pattern is likely due to the greater cumulative exposure of older animals to the PPRV [44]. Additionally, animals that were infected at an earlier age may remain seropositive for longer periods, further contributing to the higher seroprevalence in older sheep and goats [45]. In contrast [46], found a nonsignificant association between age and seropositivity for serum levels of PPRV, which might be due to the immunogenic nature of PPRV, long‐term seropositivity, and high mortality rate of highly susceptible animals. Moreover, this study revealed that sheep and goats raised in medium‐sized flocks had higher seropositivity. This observation is consistent with previous studies [47–50] where a risk factor linked to PPR was found to be flock size.

#### 4.2.1. Study Limitations

The study employed a nonprobability sampling approach, with districts purposively selected, PAs and villages conveniently selected, and animals sampled using a haphazard approach due to practical constraints associated with extensive production systems. Although necessary under field conditions, this approach may limit the representativeness and generalizability of the findings, and the estimated postvaccination antibody immunity levels should therefore be interpreted cautiously. In addition, the rapid turnover of small ruminants in the study area limited the availability of reliable animal records and prevented repeated sampling of the same animals at predefined intervals. Because prevaccination serological data were unavailable, antibodies induced by vaccination could not be clearly distinguished from those resulting from previous natural exposure, and the proportion attributable to prior PPRV exposure cannot be reliably estimated. This limitation may have influenced the interpretation of age‐related differences in serological responses as older animals may have had greater opportunities for exposure to PPRV antigens. Future longitudinal studies incorporating baseline serological assessment before vaccination would provide more accurate estimates of vaccine‐induced immunity and improve the evaluation of vaccination effectiveness under field conditions.

## 5. Conclusion and Recommendation

The study conducted in the East Dembia and Gondar Zuria districts of the North Gondar Zone, Ethiopia, provided important insights into the postvaccination antibody status of small ruminants against PPRV. The observed flock‐level antibody immunity was slightly below the 80% population immunity target recommended within the PPR‐GCES. This indicates that the vaccination program has generated substantial antibody responses among vaccinated animals but highlights the need for continued vaccination and monitoring to improve and sustain adequate population immunity. Because prevaccination serological data were unavailable, the contribution of prior natural exposure to the observed antibody responses could not be reliably quantified. Therefore, the findings should be interpreted cautiously when assessing vaccine‐induced immunity and potential PPRV exposure in the study population. The absence of significant associations between most assessed factors and PPRV antibody status suggests that additional epidemiological and operational factors may influence vaccination outcomes. These findings emphasize the importance of strengthening PVE, improving vaccination delivery systems, and maintaining effective surveillance to support Ethiopia’s progress toward PPR eradication. Based on these findings, the following recommendations are proposed:

• Regularly evaluate and modify vaccination strategies in light of local information, such as seasonal variations and animal mobility.
• The increase vaccination efforts in underserved areas and adapt strategies to local epidemiological factors, including animal movements and cross‐border threats.
• Strengthen national surveillance and postvaccination seromonitoring to track virus circulation, detect outbreaks early, and identify immunity gaps for timely adjustments as needed.

## Author Contributions

Firdyawukal Abuhay Tafere, Mastewal Birhan Atanaw, Anmaw Shite Abat, and Enyiew Alemnew Alamerew contributed to the development of the proposal, design, collection of field and lab data, formal analysis, investigation, methodology, resources, software, supervision, validation, visualization, writing up, and revising the paper. Melkie Dagnaw Fenta, Genetu Kassahun Berie, Kefale Ambachew Shiferaw, and Bemrew Admassu Mengistu contributed to conceptualization, data curation, formal analysis, investigation, methodology, resources, software, supervision, validation, visualization, writing – original draft, and writing – review and editing.

## Funding

No funding was received for this research.

## Disclosure

The final manuscript was read and approved by all the authors. Verbal informed consent to publish the image of Firdyawukal Abuhay Tafere included in this study (Annex II) was obtained before submission.

## Consent

The authors have nothing to report.

## Conflicts of Interest

The authors declare no conflicts of interest.

## Supporting Information

Additional supporting information can be found online in the Supporting Information section.

## Supporting information


**Supporting Information** The supporting annexes provide additional details relevant to the methodology and data collection of this study. Annex I: The exact, structured form we used in the field to collect data on PPR (peste des petits ruminants) risk factors. Annex II: A step‐by‐step walk through of how we collected and processed all the blood samples. Annex III: A visual guide explaining how we accurately determined the age of the sheep and goats by checking their teeth. Annex IV: A comprehensive deep‐dive into the competitive ELISA (c‐ELISA) test, including how it works, how we validated it, and how we interpreted the results. Annex V: c‐ELISA microplates used to detect specific antibodies in test sera. A positive test result is indicated by a lower optical density (OD) or color intensity in the well compared to the negative control. Results are interpreted by comparing OD values against controls and predefined thresholds. Annex VI: We obtained ethical approval for this study, which serves as evidence that the research complies with ethical standards and safeguards participant rights and welfare.

## Data Availability

Data that support the findings of this study are available in the Supporting Information of this article.
