# Supplementary material for: Serosurveillance and Postvaccination Seromonitoring of Peste Des Petits Ruminants in Small Ruminants in Central Gondar Zone, Northwest Ethiopia
Source: Transbound Emerg Dis. 2026 Sep 28;2026:3319431. doi: 10.1155/tbed/3319431 (PMC13620842; doi:10.1155/tbed/3319431)
Supplement: Supplementary file 1 [file TBED-2026-3319431-s001.docx]

**ANNEXES**

The structured sample collection format was used for this study to assess the associated risk factors of PPR prevalence.

***Annex I:*** Sample collection format

Sampling format

Zone:_______________District:______________PAs:_________village______________

GPS coordinate: Northing: ___________ Easting: __________ Elevation: ______________

| No | date | Flock size | | Spps | origin | sex | age | Vaccination status | Vaccination time | Grazingmg+ | Housing | Inter-flock con. | Introduction of NA | Animal movement |
| --- | --- | --- | --- | --- | --- | --- | --- | --- | --- | --- | --- | --- | --- | --- |
|  |  | sheep | goat |  |  |  |  |  |  |  |  |  |  |  |
| 1 |  |  |  |  |  |  |  |  |  |  |  |  |  |  |
| 2 |  |  |  |  |  |  |  |  |  |  |  |  |  |  |
| 3 |  |  |  |  |  |  |  |  |  |  |  |  |  |  |
| 4 |  |  |  |  |  |  |  |  |  |  |  |  |  |  |
| 5 |  |  |  |  |  |  |  |  |  |  |  |  |  |  |
| 6 |  |  |  |  |  |  |  |  |  |  |  |  |  |  |
| 7 |  |  |  |  |  |  |  |  |  |  |  |  |  |  |
| 8 |  |  |  |  |  |  |  |  |  |  |  |  |  |  |
| 9 |  |  |  |  |  |  |  |  |  |  |  |  |  |  |
| 10 |  |  |  |  |  |  |  |  |  |  |  |  |  |  |
| 11 |  |  |  |  |  |  |  |  |  |  |  |  |  |  |

***Annex II:*** *Blood sample collection and processing*

**Fig**.2, the figure shows collection of blood samples from small ruminants and separates serum from whole blood by using centrifugation.

**Annex III**: Small ruminant age estimation by dentition

This study was used the age estimations by dentition to categorize the age of small ruminants.

| No. of permanent incisors | | Estimated age range | |
| --- | --- | --- | --- |
| Sheep | | Goat | |
| 0 pair | Less than one year | | Under one year |
| 1 pair | 1-1½ years | | 1-2 years |
| 2 pairs | 1½-2years | | 2-3 years |
| 3 pairs | 2½-3years | | 3-4 years |
| 4 pairs | More than three years. | | More than four years |
| Broken mouth | Aged | | Aged |

Source: ESGPIP, 2009

**Annex IV** : Description, principle, validation and interpretation of c-ELISA test

**Description and Principle of the test**

The wells are coated with purified recombinant PPR N protein. The samples to be tested and control are added to the micro wells. Anti-N protein antibodies, if present form an antibody-antigen complex which masks the N protein epitopes.

An anti-N protein-Peroxidase (HRP) conjugate is added to the micro wells and fixes to the remaining free N protein epitopes, forming an antigen-conjugate-HRP complex. After washing in order to eliminate excess conjugate, the substrate solution (TMB) is added and observed for presence or absence of color development.

The resulting coloration depends on the quantities of specific antibodies present in the sample to be tested. In the absence of antibodies in the serum, a blue solution which becomes yellow after addition of the stop solution appears where as in the presence of antibodies, no coloration appears.

**Validation of the test**

According to the IDvet innovative diagnostic ID Screen® PPR competition manual, the test is validated if

- The mean OD value of the negative control (ODNC) is greater than 0.7 and
- The mean OD value of positive control (ODPC) is less than 30% of the ODNC.

**Interpretation of the test result**

Interpretation of the test result depends on competition percentage (S/N %) of the samples that is obtained by dividing OD value of the samples for ODNC multiplied by 100.

Accordingly;

- Samples with S/N % less than or equal to 50% are considered positive
- Samples with S/N% greater than 50% and less than or equal to 60% are considered doubtful
- Samples with S/N% > 60% are negative.

***Annex V****: The c-ELISA micro plates indicating the results of tested sera samples*

- Yellow-colored plate wells indicate negative results and the non-colored wells indicate positive result to PPR antibody

***Annex VI*:** Ethical Approval sheet

This study was conducted after being evaluated and approved by the Institutional Review Board of the University of Gondar with(Ref: CVMASc /UOG/RERC/22/10/2024).
